# On the Therapeutical Applications of the Solution of the Permanganate of Potash, and of Ozone

**Published:** 1864-03

**Authors:** Samuel Jackson

**Affiliations:** Emeritus Prof. of the Institutes of Medicine in the University of Pennsylvania


### Selections.

### ON THE THERAPEUTICAL APPLICATIONS OF THE SOLUTION OF THE PERMANGANATE OF POTASH, AND OF OZONE.

By SAMUEL JACKSON, M.D., Emeritus Prof. of the Institutes of Medicine in the University of Pennsylvania.

In looking over, last spring, Bouchardat's *Annuaire de Therapeutique, &c.*, for 1863, I met with the statement "*L'eau ozonisee anglaise est une dissolution de permanganate de potasse* 2, *eau* 1000," p. 95 (that the ozonized water of the English is a solution of the permanganate of potassa, in the proportion of two parts to 1000 of water).

Pincus an others had already established the disinfecting and deodorizing properties of the solution of this salt. These notices suggested to me the thought of testing its therapeutic actions and practical application. My observations were commenced in April; but confined to my office, my investigations were, of course, limited.

Having prepared the solution according to the above formula, I proceeded to ascertain its sensible properties on myself. It had no proper taste, but gave a sensation of coolness in the mouth, leaving behind a slight styptic feeling and dryness, which continued for an hour or more. Taken in the dose of a teaspoonful, slightly diluted, three times a day, it produced no prominent symptoms. It caused no inconvenience; there was some increase of appetite, which, however, was good, and an easier digestion. A diuretic action was obvious; there was no general excitement, increase of temperature, or frequency of pulse. A few days after I prescribed the solution in a case of dyspepsia, attended with loss of appetite, disordered digestion, and extreme lassitude. A teaspoonful in half a wineglass of water was directed to be taken four times a day. In a few days the patient called to report a complete recovery.

Four cases of a similar character were treated in the same manner, with a prompt and successful result.

In only one slight surgical case have I been able to test its effects. It was a foul ulcer of moderate size on the left leg, the veins being varicose. The solution was given internally, and directed to be used as a wash several times a day, walking to be avoided as much as possible, and the leg to be kept up. In a week the patient presented himself, the ulcer healthy, rapidly cicatrizing, and his appetite and digestion restored, with improved health.

The following case is of a more decided character. A young medical friend living in the country called to consult me respecting his health. He presented a complete cachectic aspect. His skin dry and cool, face pallid, no appetite, irregular digestion, very feeble, with eczema of hands, feet, and slightly on the face. I mentioned to him my experience and that of my friend Dr. F. Hinkley, Assistant-Surgeon U.S.A., at Campbell Hospital, Washington (to be noticed immediately), and asked him to give the solution of the permanganate of potash a trial. The following extracts from a letter received about a week after, gives the following results:—"A week or more has elapsed since I commenced taking the medicine you gave me, and so far as my appetite and strength are concerned, I know it has done me much good, and I shall continue to take it and give it a fair trial; it has aided my digestion and given tone to my stomach. The quantity of my blood appears to have been increased and its quality improved."

In answer to a suggestion I made in a letter to him, that he was probably too hasty in his conclusions, as the last effects mentioned could hardly have been produced in so short a time, and the facts would be better than inferences, he states, "My observations were based on the following facts, whether the time be short or long:"—

"My cheeks had more *color* in them than ever before, for, if you recollect, I have a pale-looking countenance usually. At the time of writing to you, I began to appear plethoric, and felt remarkably well for *me*, whereas, before I took the medicine I was anæmic. From the above facts, I was led to assert that it improved the quality and quantity of the blood. In regard to my present state of health, the eruption on my hands and face has almost disappeared, on which I have used the solution as a wash twice a day; but my feet, on which I have not used it, are in the same condition as they were originally, no change. Other symptoms about as they were when I wrote you first."

The following case has special interest. A gentleman advanced in life, had been affected last spring with a persistent sense of burning in the urethra, without discharge or apparent inflammation. He was under treatment by Dr. M. M. Levis, who brought him to my office. After six or seven weeks he was relieved. In September he noticed increased secretion of urine, which compelled him to rise several times in the night. In the beginning of October he called on Dr. Levis, who examined the urine. The specific gravity was 1036; it was found to contain mucus in considerable quantity. October 12th, a consultation was held at my office, when I suggested a trial with the solution of the permanganate of potassa. This was adopted, and no other remedy employed. November 5th, the Dr. called and informed me that a complete cure had been effected. The urine had gradually diminished in quantity, and was at that time entirely normal in character.

The following statement of the weekly examination of the urine was given to me by Dr. Levis:—

| | Specific gravity. | Quantity in night. | Mucus. |
|---|---|---|---|
| Oct. 12 . . | 1036 | $3\frac{1}{2}$ pints | Large amount. |
| " 19 . . | 1033 | 2 pints | Less amount. |
| " 26 . . | 1026 | $1\frac{1}{2}$ pint | Still less. |
| Nov. 4 . . | 1023 | 1 pint | A trace. |

This gentleman has since made a journey of several hundreds of miles without inconvenience or any return of the affection.

The above are the results of my own observations of the therapeutic action of this remedy. But the most remarkable and almost marvellous effects are its prompt, in most cases, its immediate action in the treatment of gangrenous wounds in the Campbell Hospital in Washington, and the United States Jarvis Hospital, Baltimore.

On the 19th of May, my young friend, Dr. F. Hinkle, of Marietta, Pa., called on me in passing through the city. He informed me he, was Acting Assist-Surgeon U.S.A., and was then stationed at Campbell Hospital, Washington. In the course of conversation on his medical and surgical experience, he mentioned the number of cases of gangrenous wounds, particularly in the wounded at the battle of Fredericksburg, the difficulty of treating them, and the ill success of the treatmnnt pursued. I informed him at once of the observations I had been making with the solution of the permanganate of potassa, and proposed to him to give it a trial. Having a conviction that ozone existed in the solution, I was strongly impressed with the belief that it would be found adapted to such cases. The Dr. at once acceded to my proposition, and obtained the salt at Mr. Blair's on leaving my office.

On the 25th of May I received a letter from him of date 24th, in which he informs me that "in reference to the treatment of hospital gangrenous wounds and gangrene, it has already proved beyond all description efficacious. In the action of the remedy you proposed I find more than I expected, and almost all I could wish. I now give you a prominent case as an illustration of its valuable effects and the instant change produced by its local application and its internal administration, upon the general character of the whole case:—

"The description of the case will be limited merely to the immediate action of the solution on the gangrenous wound. Michael Hoyau, Sergeant, Co. D, 11th Regt., Mass., aged 35; wounded May 3d, 1863, at the battle of Fredericksburg; admitted to hospital May 8. An extensive gunshot flesh wound had been received at the upper fourth of tibia and fibula of the right leg. The integuments for the space of four inches in length and three in breadth, had sloughed from gangrene, leaving at this date, May 23, the tibia exposed for three inches. The whole of the leg and up to the middle half of the thigh is infiltrated with a putrid sanious liquid and pus. The discharge is nearly a quart per diem.

"The left thigh had been penetrated by a minie ball at the commencement of the popliteal space. A considerable amount of fluid had gravitated back of the knee-joint, which was a source of great suffering. This was relieved by a counter opening giving a free discharge of the fluid. The treatment was commenced May 23, 7 A.M., at which time the situation of the patient was very critical. Pulse was thread-like and 96. Face pallid with anxious expression; head covered with cool sweat. The general temperature below the natural standard; had slept five hours in the last twenty-four. The gangrenous surface looked badly, had a dark green aspect and flabby, exuding a sanious liquid mixed with debris of dead tissues. The odor was pungent and highly offensive. The whole leg and thigh appeared as though melting into this fluid.

"The following treatment was adopted according to your suggestion:—℞.—Per mang. potassa ʒj, acid. sulph. gtt. xx, aq. comm. Oij.—M. A teaspoonful was given every three hours in a wineglass of water. The gangrenous parts were washed with the solution externally and internally, and charpie soaked in it was kept continually applied, being changed as often as the dressing became saturated with the discharge, or, when that was checked, when it became dry.

"The effects on the gangrenous tissues were instant. The flabby, sloughing and indolent surface immediately dried up, and in a few minutes presented the appearance of a wound to which a solution of nitrate of silver has been applied; or that of a delicate eschar from a slight burn, yet it gave no sensation of pain. In three hours the odor was greatly lessened, and in less than 24 hours it was barely to be perceived.

"In at least fifteen other cases of gangrene, such as of stumps of limbs, etc., its actions was no less efficacious."

The Doctor concludes:—"I am already assured that it (the solution of permang. potass) is of the greatest value in cases as above mentioned."

I received from the Doctor a communication inclosing the history of ten cases of gangrenous wounds treated in Jarvis United States Hospital, Baltimore, with the solution, and in all the gangrene was promptly arrested. He also describes the mode of application which he has found the most useful from his extended experience.

He also informs me that he is making out a report to the Surgeon-General on the permanganate of potassa and its uses. In this he will give the history of the numerous cases—I believe now nearly one hundred—of different affections in which he has employed it. A duplicate, he states, will most probably be published in the *Medical Times*, to which I refer for a full confirmation of what I predicted, from my limited experience respecting the therapeutic action of the solution of the permanganate of potash. I have a strong conviction that science has acquired in this agent a remedy of active powers, of extensive application, easily procured at a small cost, and which can be used without apprehension of risks to be incurred.

From the decided therapeutic action obtained by the practical employment of this solution, especially in the United States Hospitals by Surgeon Hinkle, it became a matter of interest to ascertain its active principles. With this view I tested it for ozone. I had prepared Scoutetten's ozonometric starch and papers. The following is his formula:—

| | |
|---|---|
| Distilled water . . . . . | 100 gram. |
| Finely-powdered starch . . . | 10 do. |
| Iodide potassium . . . . . | 1 do. |

The iodide of potassium is to be dissolved in the distilled water, and the starch powder is to be mixed in the liquid; when the mixture is finished it is to be placed in a porcelain capsule over a gentle fire, and constantly stirred with a glass rod until it assumes the pasty consistence of the domestic starch. This may be spread on paper; common letter or writing paper is to be preferred. It is then to be cut into bands and kept in a bottle or box.

I employed at first the recently prepared starch. Small portions were placed on a porcelain capsule and a few drops of the solution mixed with the test. A beautiful blue color was instantly developed, after a few minutes passing into a deep black. It compared with No. 10 the highest figure of the ozonometric scale. As ozone obtained by the usual process is not very soluble in water, olny about 6 per cent, and does not produce it reaction as rapidly or as decidedly as the permanganate solution, this last must contain a much larger proportion than could heretofore have been employed. Another difficulty was that the former solution was not permanent; it seldom retained its properties over forty-eight hours. In the first trials made I added a few drops of sulphuric acid to keep up a gentle chemical action by which ozone is generated. But this was soon found to be unnecessary, as I ascertained that the permanganate solution exposed for three to four weeks in a capsule to the air lost but little of its power on the ozonometric test.

A short account of the present state of our knowledge of oxygen and its modified or allotropic states is indispensable for the understanding of the mode of action of the solution of the permanganate. In 1842, Schœnbein discovered what he at first supposed was a new elementary body, which from its odor he named ozone. Fremy and Becquere, demonstrated it to be oxygen, the properties of which were highly intensified. This view was generally adopted. Schœnbein, from his investigations, ascertained that peroxide of $HO^2$ could not be produced from either oxygen or ozone, and he assumed hypothetically that there must exist another body in relation to ozone, which he named antozone. They are noted by the symbols $\overset{ox}{\bigcirc}$ $\overset{oz}{\ominus}$ $\overset{ant}{\oplus}$. These facts confirm the views of Mr. Faraday, that oxygen is an allotropic body, capable, like carbon, sulphur, and phosphorus, of taking on modified states, differing in their physical and chemical properties.

"The most important distinctive property of antozone $\oplus$," Schœnbein states, "is the readiness with which it unites to water, to form the peroxide of hydrogen." Ozone exists in the gaseous state, and is always existing in varying quantities in the atmosphere; it is also held in solution by water. It is formed over the surface of water, whether the immense extent of the ocean, lakes, or rivers. It is resolved or converted into passive oxygen by the various oxidizing actions it effects on effluvias, and other contaminations of the atmospheric air. It is also the active agent in bleaching and in chemical actions of the animal organism, oxygen being changed into ozone by the chemical actions in constant activity in the pulmonary tissues and capillaries during respiration.

Schœnbein was desirous to obtain antozone in an insulated or free state. With this object, he directed his attention to the set of peroxides, which he named antozonides, expecting to eliminate from them that kind or part of oxygen, he supposed to be $\oplus$ antozone. As a general result, he found "that whenever ozone $\ominus$ makes its appearance $\oplus$ antozone and its equivalent, $HO—\oplus$, are present. The rule applies also to a number of organic substances, and occurs during the electrolysis of water, never ozone without the peroxide of hydrogen, or antozonic water $HO+\oplus$."

After many unsuccessful attempts, he has obtained his object in a most unlooked-for mode, which he communicated in a letter to Mr. Faraday. A dark blue fluor spar has been long known to German mineralogists. It is remarkable by its property of yielding a peculiar and disagreeable smell when triturated. The nature and cause of this odor had never been settled. The spar occurs in a vein of granite. "A German chemist sent a specimen of it" to M. Schœnbein, "asking him to try his luck in ascertaining the nature of this smelling matter." In this attempt, he showed and proved it to be the antipode to ⊖, ozone imprisoned for thousands of years, waiting for some one to recognize and set it free. Surprising as it may seem to you, and unique as the fact certainly is, that odorous matter is my insulated antozone, which had so long baffled his researches. "How that subtle matter got into the spar, I cannot tell."*

With these facts before us, there can be but little doubt as to the composition and active principles of the solution of the permanganate of potash. Besides the salt, ozone, or active oxygen, and the peroxide of hydrogen, or, as it ought to be designated, antozonic water, HO+⊕, are also present. The last two are bodies endowed with most active chemical properties, which are brought into action on the decomposing organic structure in gangrenous wounds. The disorganizing process is arrested, the organic or vital actions of the surrounding tissues, reduced to the lowest ebb, are roused into activity, and the fluids are renovated by the exciting and oxygenating properties of ozone and oxygenated or antozonic water. The essential conditions of vital organizing reaction are in this mode locally renewed, and a healing process established.

Taken internally, it enters the blood, and excites the molecular or chemical action of that fluid—an indispensable condition of life. The cessation of those actions is immediate death—it is the mode by which carbonic oxide occasions nearly instant death. These exciting agents, it is most probable, produce also arterial tension, now demonstrated to be the regulator of the capillary or nutritive and vital circulation.

We have the evidence of Gorup Besanez that ozone exerts a kindred action on organic substances. Albumen and casein are changed into products similar to those effected by digestion. Fibrin, it is stated, resisted its action. Should this fact be confirmed, it would show some special chemical distinction between it and albumen, contrary to the observations of M. Cl. Bernard and other physiologists, who regard them as mere transient states of the same substances mutually passing into each other. It had little or no action on urea, allantoin, kreatin, sugar of milk, and hippuric acid—bodies of more stable composition. Bile absorbed ozone and lost its color; its mucus was decomposed.

As bromine has been found very successful by Dr. Goldsmith and some of our surgeons, in the treatment of gangrenous wounds, it occurred to me that probably its solution in water

* Philosophical Magazine, 4th series, Vol. XXI., p. 88.

might develop ozone and antozone similar to the permanganate of potash. I procured a solution of gr. xv to ℥ij of water. With the ozone test it instantly struck a fine blue color, which in a few minutes became of a deep black. From the volatility of bromine great care is required to preserve it for any length of time. The above proportion of bromine is far too strong for internal use. A formula proposed by Ozanam in 1860, makes a preparation that is permanent, and may be employed internally. It is as follows:—

| | | |
|---|---|---|
| Bromine, pure . . . | 10 centigram. | 2 drops. |
| Bromide of potassium . . | 10 do. | |
| Distilled water . . . | 100 gram. | |

M.

This solution gives a deep blue with the ozone test, which soon passes into black.

From the similarity between bromine and chlorine, I was led to test the latter with the ozone test. A drachm of chloride of lime was put into a pint bottle and a few drops of sulphuric acid aided; in a few moments the bottle was filled with chlorine. A dried test paper was then introduced; it assumed in a few moments a bluff color; when the paper was moistenedit instantly changed into the blue color, which soon became black. When the moist paper was introduced these changes took place in the shortest period.

The test paper acted on by the solutions of permanganate of potash, bromine, and chlorine exhibited equal intensity of color; they could not be distinguished.

These facts appear to indicate ozone or antozone as the active principle of the solution of bromine as well as of the permanganate of potash and chlorine.

